# Nomogram based on MRI for preoperative prediction of Ki-67 expression in patients with intrahepatic mass cholangiocarcinoma

**DOI:** 10.1007/s00261-022-03719-7

**Published:** 2022-11-19

**Authors:** Xiang Chen, Jingfen Zhu, Zigui Zou, Mingzhan Du, Junjian Xie, Yujie Ye, Ling Zhang, Yonggang Li

**Affiliations:** 1 Department of Radiology, The First Affiliated Hospital of Soochow University, Shizi Street 188#, Suzhou, 215000 Jiangsu People’s Republic of China; 2 Department of Pathology, The First Affiliated Hospital of Soochow University, Suzhou, 215000 Jiangsu People’s Republic of China; 3 Department of Radiology, Sun Yat-Sen University Cancer Center, Dongfeng East Road 651#, Guangzhou, 510060 Guangdong People’s Republic of China; 4 State Key Laboratory of Oncology in South China, Guangzhou, 510060 Guangdong People’s Republic of China; 5 Collaborative Innovation Center for Cancer Medicine, Guangzhou, 510060 Guangdong People’s Republic of China; 6 National Clinical Research Center for Hematologic Diseases, The First Affiliated Hospital of Soochow University, Suzhou, 215000 Jiangsu People’s Republic of China; 7 Institute of Medical Imaging, Soochow University, Suzhou, 215000 Jiangsu People’s Republic of China

**Keywords:** Cholangiocarcinoma, Ki-67 expression, Magnetic resonance imaging, Prognosis, Liver neoplasms

## Abstract

**Objectives:**

To validate a new nomogram based on magnetic resonance imaging (MRI) for pre-operative prediction of Ki-67 expression in patients with intrahepatic mass cholangiocarcinoma (IMCC).

**Methods:**

A total of 78 patients with clinicopathologically confirmed IMCC who underwent pre-operative gadolinium-ethoxybenzyl-diethylenetriamine pentaacetic acid enhanced MRI between 2016 and 2022 were enrolled in the training and validation group (53 patients and 25 patients, respectively). Images including qualitative, quantitative MRI features and clinical data were evaluated. Univariate analysis and multivariate logistic regression were used to select the independent predictors and establish different predictive models. The predictive performance was validated by operating characteristic curve (ROC) analysis, calibration curve, and decision curve analysis (DCA). The validation cohort was used to test the predictive performance of the optimal model. The nomogram was constructed with the optimal model.

**Results:**

In the training cohort, independent predictors obtained from the combined model were DWI (OR 1822.741; 95% CI 6.189, 536,781.805; *P* = 0.01) and HBP enhancement pattern (OR 14.270; 95% CI 1.044, 195.039; *P* = 0.046). The combined model showed the good performance (AUC 0.981; 95% CI 0.952, 1.000) for predicting Ki-67 expression. In the validation cohort, The combined model (AUC 0.909; 95% CI 0.787, 1.000)showed the best performance compared to the clinical model (AUC 0.448; 95% CI 0.196, 0.700) and MRI model (AUC 0.770; 95% CI 0.570, 0.970).

**Conclusion:**

This new nomogram has a good performance in predicting Ki-67 expression in patients with IMCC, which could help the decision-making of the patients’ therapy strategies.

**Graphical abstract:**

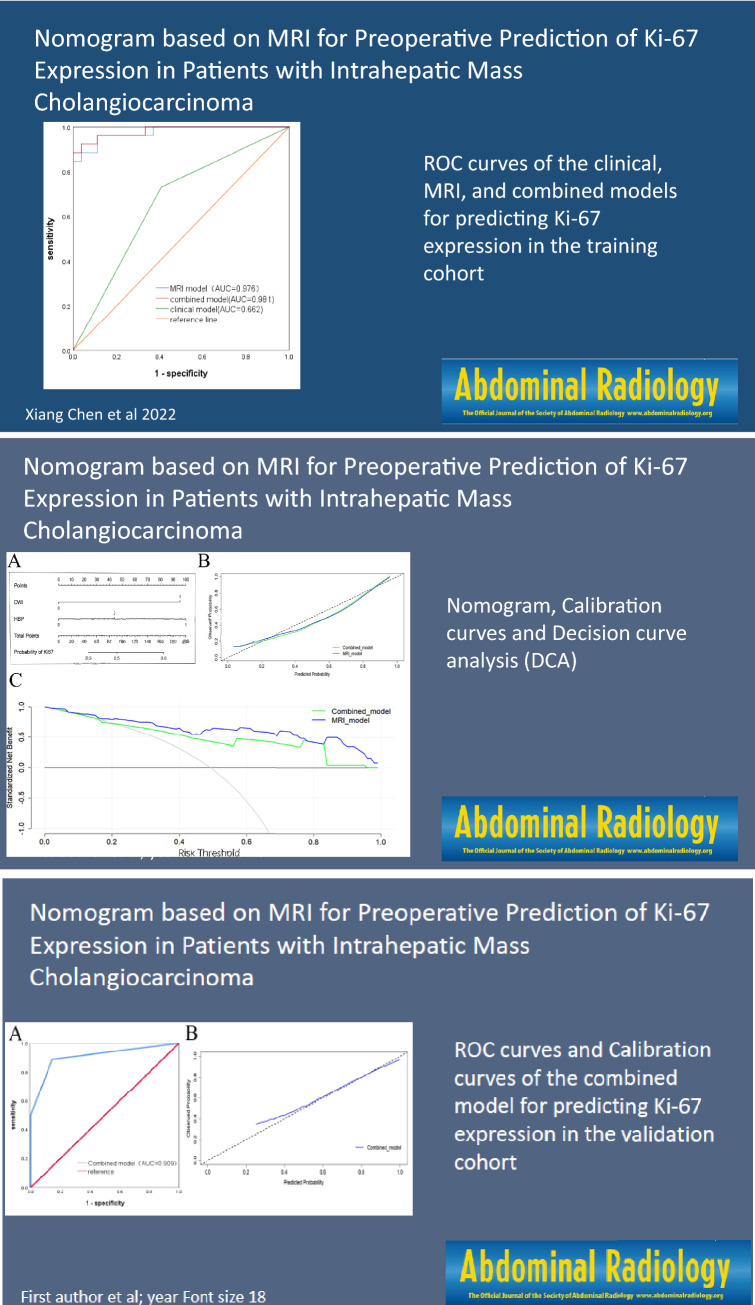

**Supplementary Information:**

The online version contains supplementary material available at 10.1007/s00261-022-03719-7.

## Introduction

Cholangiocarcinoma was the most common liver malignancy after hepatocellular carcinoma (HCC). Intrahepatic cholangiocarcinoma (ICC) arises from the peripheral bile ducts within the liver parenchyma and accounts for 10% of all cholangiocarcinomas. Over the past 40 years, the overall incidence of cholangiocarcinoma has increased year by year [1].

The ICC presents three growth patterns based on the gross appearance: mass-forming (MF), periductal infiltrating (PI), and intraductal growing (IG) [2, 3]. Intrahepatic mass-forming cholangiocarcinoma (IMCC) is the most common type of ICC, accounting for about 65% of all ICCs cases [4]. The imaging features of IMCC are an irregularly shaped solid mass with peripheral rim enhancement and incomplete concentric pooling of contrast material on dynamic contrast material–enhanced computed tomography (CT) or magnetic resonance imaging (MRI) [5, 6]. Surgery is a first-line treatment approach in patients with IMCC. However, the 5-year recurrence-free survival rate and 5-year overall survival (OS) after surgery were 2–39% and 5–56%, respectively [7–10]. Recent studies have shown that Ki-67 expression is an independent predictor of cancer progression [8]. Qiang et al*.* reported that Ki-67 expression could be used to assess the biological behavior and prognosis of the ICC [11]. However, current methods to assess Ki-67 expression rely basically on immunohistochemical examination and postoperative pathology. Therefore, assessing Ki-67 expression prior to surgery or prior to the decision to have surgery may help the selection of appropriate treatment, improving remission rates.

MRI has been used for predicting Ki-67 expression in hepatocellular carcinoma and breast cancer, with a high diagnostic value [12–14]. However, there are no studies exploring the predictive value of the MRI to predict the Ki-67 expression in patients with IMCC. In this study, we established and validated a nomogram model that combines MRI features and clinical data for pre-operative prediction of Ki-67 expression in patients with IMCC. An accurate pre-operative prediction of Ki-67 expression level could be useful in developing appropriate treatments, particularly for those patients with unresectable IMCC.

## Material and methods

The institutional review boards of two institutions, Site 1 (The First Affiliated Hospital of Soochow University) and Site 2 (Sun Yat-sen University Cancer Center) approved this retrospective study and waived the requirement for written informed consent.

### Patients

**Fig. 1:**
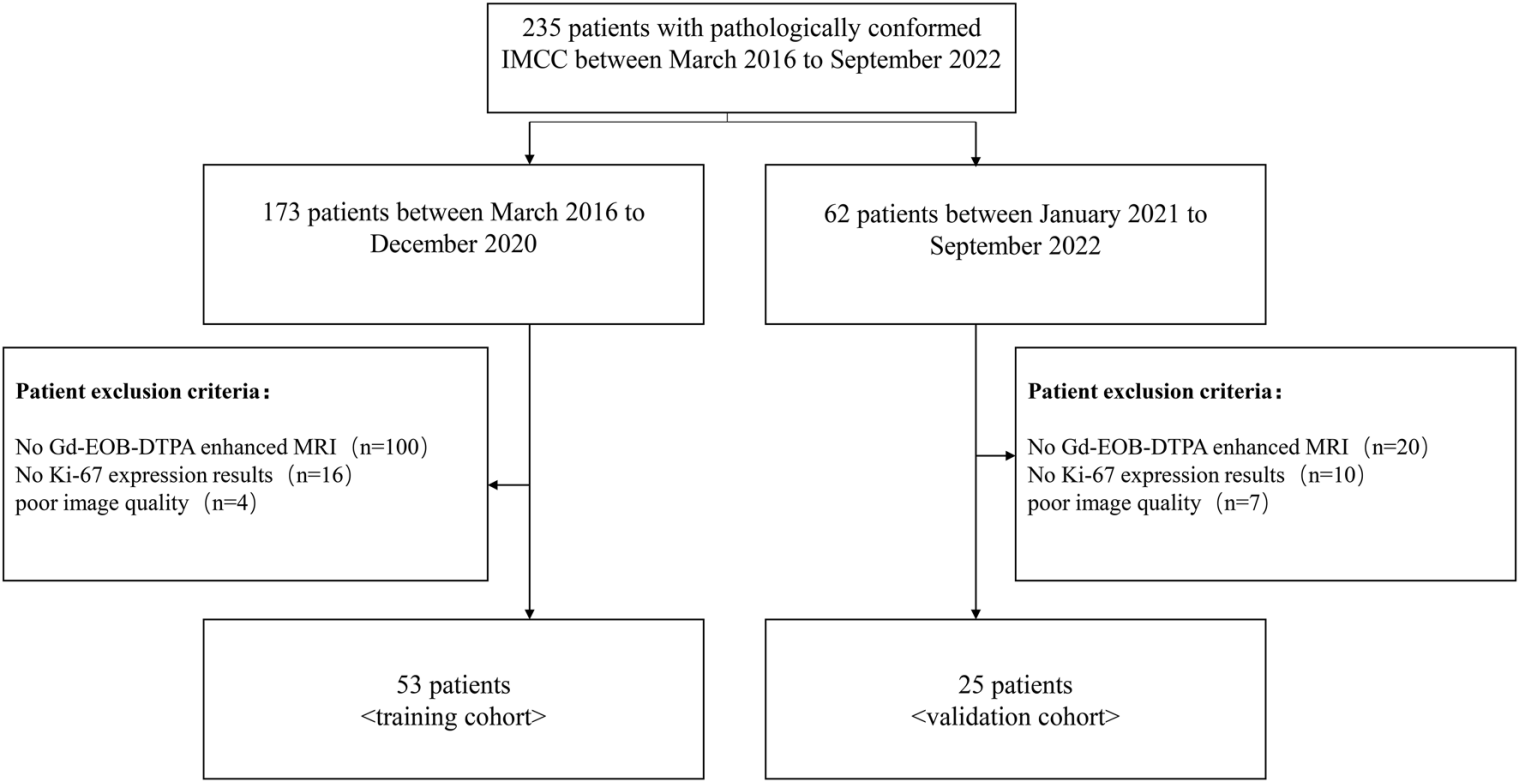
Study flow chart of the training and validation cohorts. *IMCC* Intrahepatic mass cholangiocarcinoma; *Gd‐EOB‐DTPA* gadolinium-ethoxybenzyl-diethylenetriamine pentaacetic acid

Between March 2016 and September 2022, 235 patients with a pathologic diagnosis of IMCC were retrospectively identified through electronic medical records. Those who met the following inclusion criteria were selected: (1) patients with a pathologically confirmed IMCC (2) those with positive immunohistochemical Ki-67 expression results based on surgical resection (*n* = 212) or percutaneous biopsy (*n* = 23) (3) gadolinium-ethoxybenzyl-diethylenetriamine penta acetic acid (Gd‐EOB‐DTPA) enhanced MRI performed within 2 weeks before surgery. Exclusion criteria was: (1) No Gd-EOB-DTPA enhanced MRI (2) No Ki-67 expression results (3) poor image quality. Figure 1 depicted the patient selection and grouping process.

### Clinical data

Information about chronic hepatitis, cirrhosis, Child–Pugh score, schistosomiasis, alpha-fetoprotein (AFP), carcinoembryonic antigen (CEA), carbohydrate antigen 19–9 (CA19-9) were obtained through the electronic medical records system.

### Histopathological analysis

**Fig. 2:**
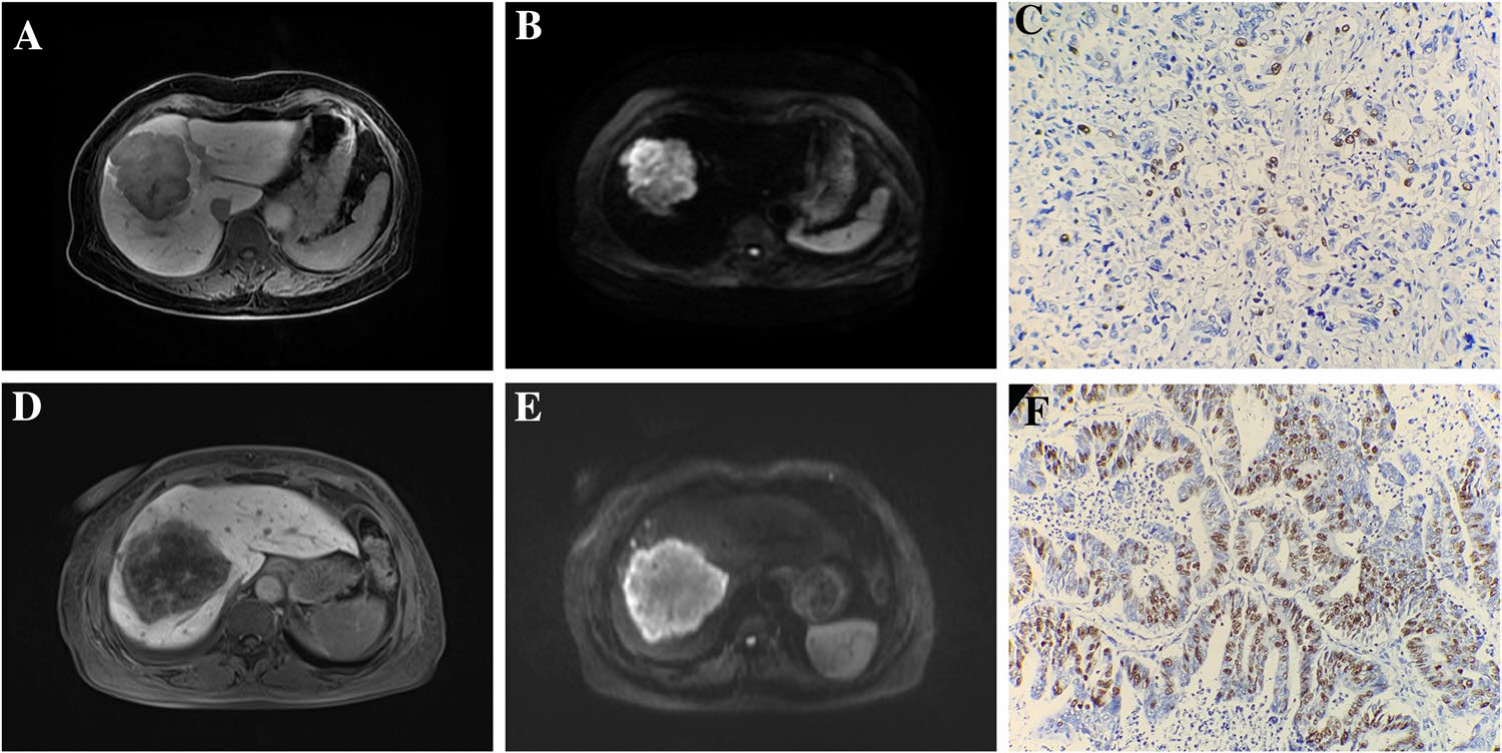
MRI images and pathological control. (**A–C**) A 67 years old female patient with low Ki-67 expression (about 15%). **A** Cloud sign on HBP; **B** diffusely hyperintense on DWI; **C** histologic variations (immunohistochemical staining, × 200-fold). (**D–F**) A 71 years old female patient with high Ki-67 expression (about 45%). **D** partially hyperintense on HBP; **E** target sign on DWI; **F** histologic variations (immunohistochemical staining, × 200-fold)

The histopathological examination was performed by two experienced pathologists who were blinded to radiological and clinical results. Immunohistochemical staining was used to ensure Ki-67 expression. Ten fields of view were selected under high magnification (× 400), and 100 cells were counted in each field of view (in the hot spots within the tumor). Positive Ki-67 expression was the presence of tan (yellow) colored particles in the nucleus. The labeling index of Ki-67 expression was evaluated by the positive percentage in total cells. According to the index of Ki-67 expression, immunoreactive cells were divided into the low (< 30% immunoreactivity) and high-expression groups (≥ 30% immunoreactivity) [15] (Fig. 2C, F).

### MRI protocol

Because of the retrospective nature of the study, a variety of MR imaging units were used, including 3.0 T MRIs (Siemens Magnetom Verio 3.0 T; Siemens Magnetom Skyra 3.0 T; GE Signa HDxt 3.0 T) with a dedicated phased-array body coil. The scan sequence included: in-phase and out-of-phase T1 weighted imaging; T2 weighted fat-suppression turbo spin-echo sequence;diffusion-weighted imaging (DWI), ADC maps, and dynamic 3D T1WI contrast-enhanced imaging. All pulse sequence parameters are shown in Supplementary Appendix 1.

### Image analysis

#### Qualitative MRI analysis

All MRI examinations were evaluated in consensus by two senior radiologists (observer 1 and observer 2, both with 9 years of experience in abdominal MRI). In case of disagreement, a consensus was reached by discussion. All images were evaluated by a picture archiving and communication system (Neusoft PACS/RIS V5.5), and both radiologists were blind to clinical, laboratory, and pathological information. The largest lesion was evaluated when multiple lesions were present in the liver. The qualitative features of MRI mainly included signal intensity of T1-weighted imaging (T1WI), T2-weighted imaging (T2WI), diffusion-weighted imaging (DWI), arterial phase enhancement pattern, dynamic enhancement pattern, HBP enhancement pattern, morphological and auxiliary features. T1WI and T2WI were divided into hypointensity, isointensity, and hyperintensity. The signal intensity of the lesion on T1WI and the T2WI was the signal of maximum level compared with the surrounding normal liver parenchyma (visual observation). DWI included: (1) diffusely hyperintense: areas of hyperintense in more than one-third of the tumor; (2) target sign: areas of hyperintense restriction in less than one-third of the tumor (Fig. 2B, E) [16]. Arterial phase enhancement patterns included: (1) peripherally hyperintense: < 10% of the largest tumor diameter is hyperintense; (2) partially hyperintense: 10–70% of the largest tumor diameter is hyperintense; (3) diffusely hyperintense: more than 70% of the largest tumor diameter is hyperintense (Supplementary Fig. 1A–C) [7]. Dynamic enhancement patterns mainly included three types: (1) progressive CE (contrast enhancement): the nodule is enhanced progressively over time, reaching maximal intensity in delayed phases; (2) stable, persistent CE: the nodule enhancement remains invariable through the AP and EP; (3) washout, intense contrast uptake during the AP followed by contrast washout in delayed phases. The hepatobiliary phase (HBP) enhancement pattern was divided into three types: (1) cloud sign: a central hyperintense area with a peripheral hypointense rim defect, > 90% of the largest tumor diameter is hyperintense; (2) partially hyperintense: 10–90% of the largest tumor diameter is hyperintense (Supplementary Fig. 1D–F); (3) peripherally hyperintense: < 10% of the largest tumor diameter is hyperintense. Auxiliary signs included: (1) halo signs, defined as peripherally hyperintense around lesions in the arterial phase; (2) peritumoral hypointense on HBP enhancement pattern, defined as low signal around the lesion on HBP; (3) abnormal perfusion, defined as irregularly abnormal hyperintense around the lesions or other parts on arterial phase.

#### Quantitative MRI analysis

Quantitative analysis was performed by two radiologists using MicroDicom (3.9.5.666). The quantitative analysis of MRI mainly included diameter, ADC value, and the signal ratio of lesion-liver parenchyma on HBP (SIR-HBP). The diameter was measured by each radiologist at the maximum axial image on HBP. Regions of interest (ROIs) computed the signal intensity (SI) on ADC, including almost the entire area of the homogeneous part of the tumor, avoiding the outermost part to exclude a partial volume effect of adjacent normal liver tissue. ROIs in the normal liver parenchyma selected a size of > 1 cm^2^. ADC_mean_ values of the lesions were calculated using monoexponential fitting of the SI decay curve with the following formula using two *b* values: ADC = ln (S2/S1)/(b1-b2), where S1 and S2 were the signal intensities at *b* values b1 0 or 50 and b2 800 s/mm^2^, respectively. Normalized ADC values for ADCmean were calculated using ADC values of liver parenchyma as reference tissue. A normalized ADC (nADC) was defined relative to the liver parenchyma as the ratio ADC_tumor_/ADC_liver_ [17, 18]. Regions of interest (ROIs) were measured maximal cross-sectional area of the lesions and at the adjacent upper, and lower level on the HBP axial image, and then the average SIR-HBP was taken. The same method was used to measure the normal live parenchymal signal intensity. The size and position of each ROI were consistent, and bile ducts, blood vessels, bleeding, cysts, and necrosis were avoided. ROIs of the lesions were measured 3 times and the average was recorded. Based on these quantitative measurements, the signal ratio of lesion-liver parenchyma on HBP (SIR-HBP) was defined as follows: HBP (SIR-HBP) = SI_lesion_/SI_liver_.

### Statistical analysis

The inter reader agreement for any of the qualitative MRI features was assessed by the kappa statistic. The value of kappa statistic > 0.75 indicated good consistency. The intraclass correlation coefficients (ICCs) were used to assess the agreement of diameter, ADC, and SIR-HBP by two radiologists. An ICC > 0.75 indicated good consistency. The Independent t-test or Mann–Whitney *U* test was used for the continuous variables, and either the χ2 test or Fisher exact test was used for categorical variables when appropriate. Significant variables (*P* < 0.05) in univariate analysis for predicting Ki-67 expression were used as covariates in multivariate logistic regression analysis for establishing a clinical model, MRI model, and combined model, respectively. The Delong test was applied to compare the area under the curve (AUC) between different models. The predictive performances were validated by receiver operating characteristic curve (ROC) analysis, calibration curve, and decision curve analysis (DCA). Based on the AUC comparison results, the nomograph of the optimal model for predicting Ki-67 expression was constructed. For the validation cohort, the area under curve (AUC) and calibration curve were used to validate. A *P* value of < 0.05 were considered to be statistically significant. ROC analysis was performed by SPSS Statistics version 26.0 and Delong test by MedCalc Statistical Software version 20.0. the "rms" "DecisionCurve" packages in the R software version 4.1.2.

## Results

### Clinical and pathologic characteristics

Clinicopathologic characteristics of IMCC patients in the training and validation cohorts are shown in Table 1. 78 patients (mean age, 60.17 ± 9.97 years; range, 39–85 years) with IMCC, including 49 men (mean age, 58.94 ± 10.96 years; range, 39–85 years) and 29women (mean age, 62.24 ± 7.75 years; range, 45–73 years) were analyzed. There was no statistically significant difference between the training and validation cohorts (*P* > 0.05).

**Table 1:** Clinicopathologic characteristics of IMCC patients in the training and validation cohorts

Characteristics	Training cohort (*n* = 53)			Validation cohort (*n* = 25)	*P* value
	Low expression (*n* = 27)	High expression (*n* = 26)	*P* value		
Age (year)	60.70 ± 1.94	61.31 ± 1.83	0.650	58.40 ± 10.56	0.285
Gender			0.646		1.000
Male	16 (59.3)	17 (65.4)		16 (64.0)	
Female	11 (40.7)	9 (34.6)		9 (36.0)	
Chronic hepatitis			0.018		0.535
Negative	11 (40.7)	19 (73.1)		16 (64.0)	
Positive	16 (59.3)	7 (26.9)		9 (36.0)	
Cirrhosis			0.337		0.059
Negative	8 (29.6)	5 (19.2)		24 (96.0)	
Positive	19 (70.4)	21 (80.8)		1 (4.0)	
Child–Pugh score			0.317		0.153
Child–Pugh class A	21 (77.8)	17 (65.4)		22 (88.0)	
Child–Pugh class B	6 (22.2)	9 (34.6)		3 (12.0)	
Child–Pugh class C	0 (0.0)	0 (0.0)		0 (0.00)	
Schistosomiasis			0.491		0.541
Negative	27 (0.0)	25 (96.2)		24 (96.0)	
Positive	0 (100.0)	1 (3.8)		1 (4.0)	
AFP			0.469		0.522
< 20 μg/L	24 (88.9)	21 (80.8)		19 (76.0)	
> 20 μg/L	3 (11.1)	5 (19.2)		6 (24.0)	
CEA			0.491		0.180
< 5 ng/mL	20 (74.1)	17 (65.4)		21 (84.0)	
> 5 ng/mL	7 (25.9)	9 (34.6)		4 (16.0)	
CA19-9			0.213		0.165
< 37u/mL	15 (55.6)	10 (38.5)		16 (64.0)	
> 37u/mL	12 (44.4)	16 (61.5)		9 (36.0)	

*IMCC* Intrahepatic mass cholangiocarcinoma, *AFP* alpha-fetoprotein, *CEA* carcinoma embryonic antigen, *CA19-9* carbohydrate antigen 19–9

### Qualitative MRI analysis

The inter reader agreement for any of the qualitative MRI features was excellent (The value of kappa statistic > 0.75) (Supplementary Appendix 2).The morphologic features and auxiliary findings in the low- and high-expression groups are shown in Table 2. There was no significant difference between the two groups in location, margin, contour, satellite lesions, intrahepatic metastasis, biliary dilation, capsular retraction, and ascites (*P* > 0.05).

**Table 2:** IMCC morphologic appearance and ancillary findings at qualitative MRI review

Characteristics	Ki-67 (*n* = 53)		*P* value
	Low expression (*n* = 27)	High expression (*n* = 26)	
Location			0.601
Right lobe	14 (51.9)	11 (42.3)	
Left lobe	6 (22.2)	9 (34.6)	
Right and left lobe	7 (25.9)	6 (23.1)	
Margin			0.704
Sharp	24 (88.9)	22 (84.6)	
Indistinct	3 (11.1)	4 (15.4)	
Contour			1.000
Nodular	11 (40.7)	11 (42.3)	
Lobulated	13 (48.1)	12 (46.2)	
Irregular	3 (11.1)	3 (11.5)	
Satellite lesions			0.685
Positive	12 (44.4)	13 (50.0)	
Negative	15 (55.6)	13 (50.0)	
Intrahepatic metastasis			0.487
Positive	13 (48.1)	15 (57.7)	
Negative	14 (51.9)	11 (42.3)	
Biliary dilation			0.318
Positive	13 (48.1)	9 (34.6)	
Negative	14 (51.9)	17 (65.4)	
Capsular retraction			0.884
Positive	13 (48.1)	12 (46.2)	
Negative	14 (51.9)	14 (53.8)	
Ascites			0.497
Positive	19 (70.4)	16 (61.5)	
Negative	8 (29.6)	10 (38.5)	

*IMCC* intrahepatic mass cholangiocarcinoma, *MRI* Magnetic Resonance Imaging

Yet, on MRI enhancement sequences, the dynamic enhancement pattern and HBP enhancement pattern for predicting Ki-67 expression were different (*P* < 0.001, *P* < 0.001) (Table 3); the low-expression group had a more progressive CE (15/27, 55.5%), while the high-expression group had a more stable, persistent CE (18/26, 69.2%). The most common HBP enhancement pattern in the low-expression group of Ki-67 was cloud sign (20/27, 74.1%), while partially hyperintense was mainly seen in the high-expression group (19/26, 73.1%).

**Table 3:** IMCC imaging findings at qualitative MRI review

Characteristics	Ki-67 (*n* = 53)		*P* value
	Low expression (*n* = 27)	High expression (*n* = 26)	
Arterial phase enhancement pattern			0.082
Peripherally hyperintense	11 (40.7)	18 (69.2)	
Partially hyperintense	7 (25.9)	5 (19.2)	
Diffusely hyperintense	9 (33.3)	3 (11.5)	
Dynamic enhancement pattern			< 0.001
Progressive CE	15 (55.6)	7 (26.9)	
Stable, persistent CE	3 (11.1)	18 (69.2)	
Washout	9 (33.3)	1 (3.8)	
HBP enhancement pattern			< 0.001
Cloud sign	20 (74.1)	5 (19.2)	
Partially hyperintense	6 (22.2)	19 (73.1)	
Peripherally hyperintense	1 (3.7)	2 (7.7)	
Abnormal perfusion			0.344
Positive	7 (25.9)	4 (15.4)	
Negative	20 (74.1)	22 (84.6)	
Halo sign			0.068
Positive	2 (7.4)	8 (30.8)	
Negative	25 (92.6)	18 (69.2)	
Peritumoral hypointense on HBP			0.678
Positive	13 (48.1)	14 (53.8)	
Negative	14 (51.9)	12 (46.2)	
T1WI			1.000
Hypointense	26 (96.3)	26 (100.0)	
Isointense	1 (3.7)	0 (0.0)	
Hyperintense	0 (0.0)	0 (0.0)	
T2WI			0.491
Hypointense	0 (0.0)	0 (0.0)	
Isointense	0 (0.0)	1 (3.8)	
Hyperintense	27 (100.0)	25 (96.2)	
DWI			< 0.001
Diffusely hyperintense	22 (81.5)	3 (11.5)	
Target sign	5 (18.5)	23 (88.5)	

*IMCC* intrahepatic mass cholangiocarcinoma, *MRI* Magnetic Resonance Imaging, *T1WI* T1-weighted imaging, *T2WI* T2-weighted imaging, *CE* contrast enhancement, *HBP* hepatobiliary phase, *DWI* diffusion-weighted imaging

In the conventional sequences, the difference in DWI between the low and the high-expression group predicting Ki-67 expression was statistically significant (*P* < 0.001). The most common performance of DWI in the low-expression group of predicting Ki-67 expression was diffusely hyperintense (22/27, 81.5%), while in the high-expression group was target sign (23/26, 88.5%). Other features showed no statistically significant differences (*P* > 0.05).

### Quantitative MRI analysis

The quantitative MRI features of the IMCCs are presented in Table 4. The agreements of diameter, SIR-HBP, ADC_mean_, and the nADC_mean_ between two readers were excellent (interclass correlation coefficient > 0.75) (Supplementary Appendix 3). Fifty-three patients had a mean diameter of 6.33 ± 0.42 cm (range 1.99–14.40 cm); in the low-expression group, the mean diameter was 3.69 cm (range 3.12–6.31 cm) while it was 7.80 cm (range 5.81–9.85 cm) in the high-expression group (*P* < 0.001).

**Table 4:** Quantitative MRI findings related to Ki-67 expression

Characteristics	Ki67 (*n* = 53)		*P* value
	Low expression (*n* = 27)	High expression (*n* = 26)	
Diameter(cm)	3.69 (3.12, 6.31)	7.96 (5.81, 9.85)	< 0.001
SIR-HBP	0.61 ± 0.03	0.51 ± 0.04	0.047
ADCmean (× 10^−3^mm^2^/s)	1.33 (1.16–1.50)	1.47 (1.20–1.70)	0.102
nADCmean (× 10^−3^mm^2^/s)	0.03 (0.01– 0.07)	0.046 (0.02–0.09)	0.240

*MRI* Magnetic Resonance Imaging, *SIR-HBP* Signal ratio of foci to liver parenchyma on HBP, *ADC* apparent diffusion coefficient, *ADCmean* ADC values of the lesions, *nADCmean* A normalized ADC (nADC) was defined relative to the liver parenchyma as the ratio ADCtumor/ADCliver.

Moreover, 53 patients had a mean SIR-HBP of 0.56 ± 0.27 (range 0.19–0.97); in the low-expression group, the mean SIR-HBP was 0.61 ± 0.03 (range 0.23–0.97), while in the high-expression group it was 0.51 ± 0.04 (range 0.19–0.84) (*P* < 0.001).

### Diagnostic performance and comparison of different models

**Fig. 3:**
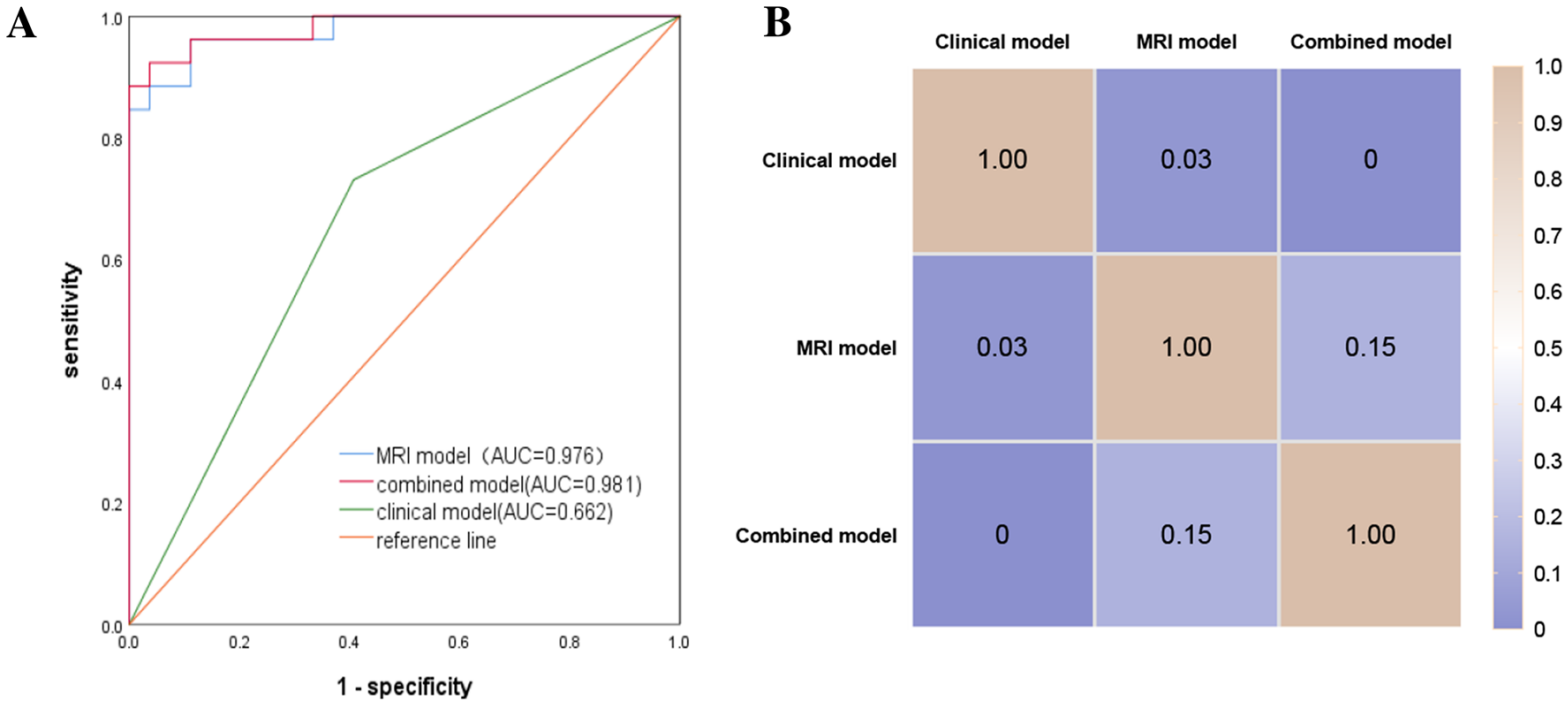
**A** ROC curves of the clinical, MRI, and combined models for predicting Ki-67 expression in the training cohort; **B** Heatmap comparison of the models for predicting Ki-67 expression in the validation cohort

The result of multivariate logistics for predicting Ki-67 expression are shown in Supplementary Appendix 4. The clinical model includes chronic hepatitis as an independent predictor, while the MRI model includes diameter and DWI, and the combined model includes DWI and HBP enhancement pattern. The combined model for predicting Ki-67 expression had the best AUC of 0.981 on the training cohorts (Fig. 3A). The AUC of the clinical model(AUC 0.448; 95%CI 0.196, 0.700) was statistically different(all *P* < 0.05)compared to the MRI model (AUC 0.770; 95% CI 0.570, 0.970)and combined model(AUC 0.909; 95% CI 0.787, 1.000), while there was no difference (*P* = 0.15)in AUC between the MRI model and the combined model in the validation cohort(DeLong test). A comparison of the different models is shown as a heat map in Fig. 3B.

### Training and validation of the predictive nomogram

**Fig. 4:**
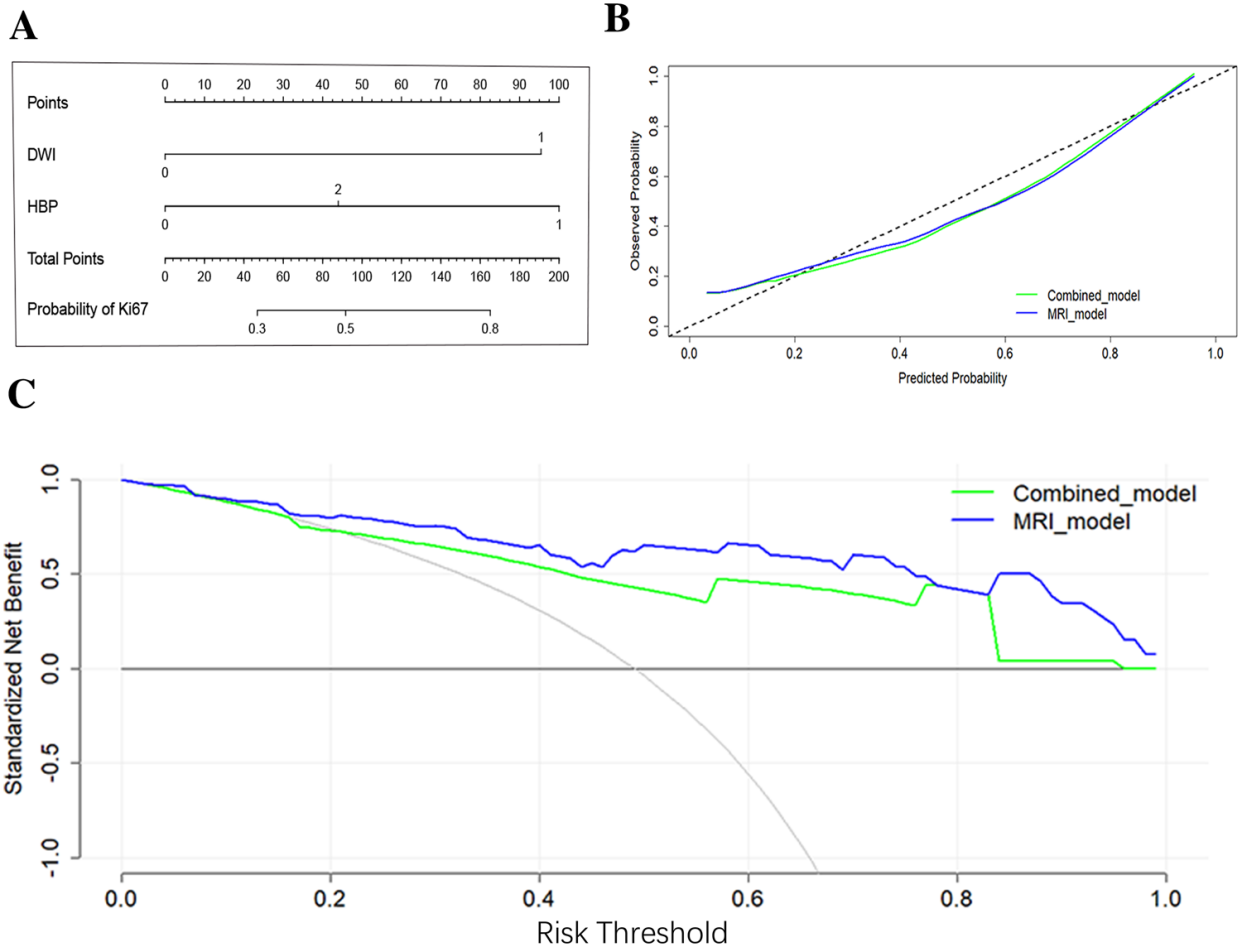
**A** Nomogram of the combined model for the prediction of Ki-67 expression. DWI, diffusion-weighted imaging; 0, diffusely hyperintense; 1, target sign; *HBP* hepatobiliary phase; 0, cloud sign; 1, partially hyperintense signal; 2, peripherally hyperintense signal; **B** Calibration curves of the nomogram for different models with predicting Ki-67 expression; **C** Decision curve analysis (DCA) of different models with predicting Ki-67 expression

**Fig. 5:**
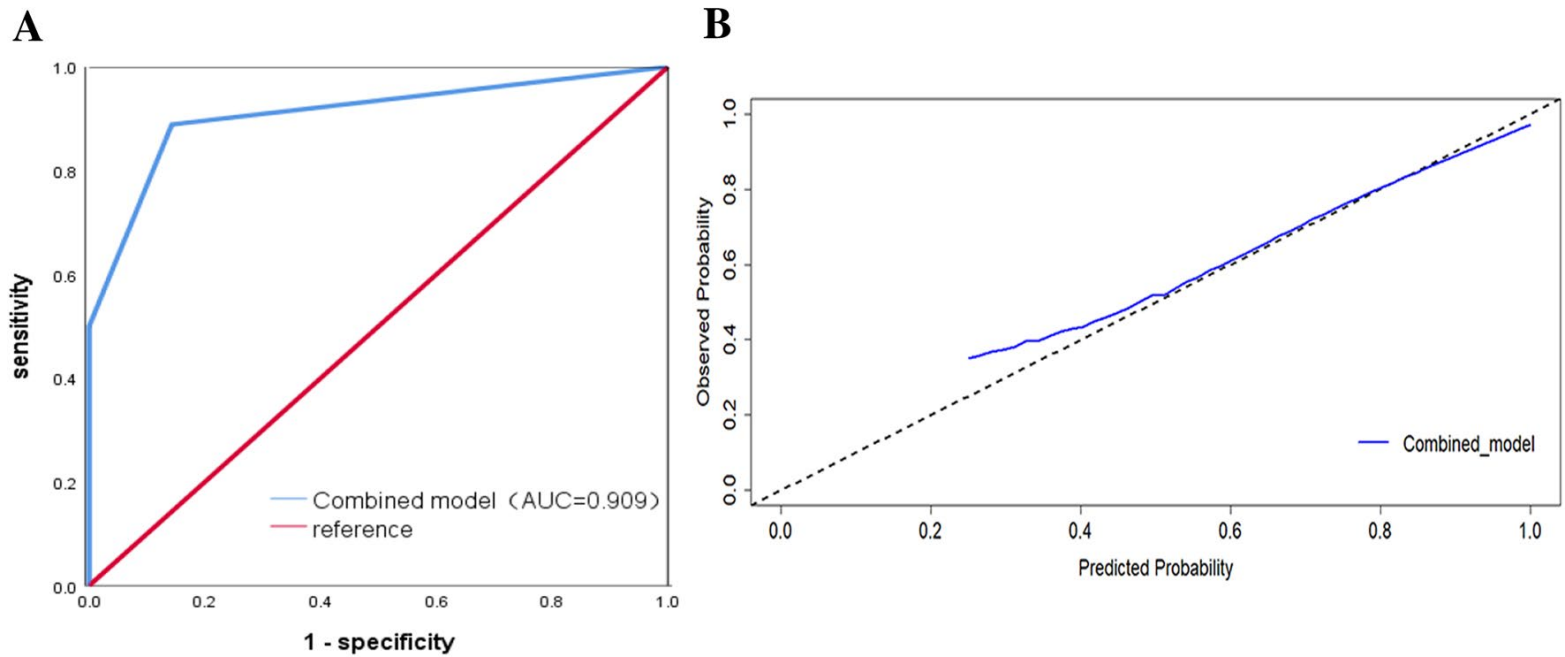
**A** ROC curves of the combined model for predicting Ki-67 expression in the validation cohort; **B** Calibration curves of the combined model for predicting Ki-67 expression in the validation cohort

The combined model for predicting Ki-67 expression was further used to construct a nomogram figure (Fig. 4A). For the calibration curve of predicting Ki-67 expression, the Hosmer–Lemeshow fitting test indicated that the nomogram for different models had a good fit (*P* = 0.942, *P* = 0.881) (Fig. 4B). With a threshold probability of 10–99%, the decision curve graph showed that the combined model basically had consistent predictive performance with the MRI model (Fig. 4C). The validation cohort confirmed the reliability and stability of the combined model, which achieved an AUC of 0.909 for predicting Ki-67 expression (95% CI 0.787, 1.000) (Fig. 5A, B).

## Discussion

Some researchers have proposed using prediction nomograms or statistical cure modeling in patients with IMCC [19, 20]. However, all of the aforementioned staging systems or prediction models are based on demographic and clinical-pathologic characteristics that are acquired after surgery. Adoxetic acid-enhanced MRI is a leading method for the evaluation of hepatic malignancies. Gadoxetic acid functions as both an extracellular agent and a hepatocyte-selected agent.

Ki-67 expression is a significant biomarker of prognosis in patients with IMCC. However, Ki-67 expression of IMCC is mainly evaluated by immunohistochemical examination and its procedure is invasive and obtained postoperatively. In this study, we developed and validated a nomogram consisting of DWI data and HBP for predicting Ki-67 expression. The result showed that the nomogram had a good performance in predicting Ki-67 expression in both the training cohort and validation cohort, which indicated that non-invasive pre-operative MRI could provide a valuable prognostic marker to assist physicians in making a more rational treatment strategy for patients not eligible for surgical procedure.

DWI and HBP were independent predictors of predicting Ki-67 expression in IMCC. DWI is a form of MR imaging based on random Brownian motion of water molecules within a voxel of tissue that reflects the tumor microenvironment based on the cellular density and architectural change [21, 22]. One of ICC's notoriously characteristic histologic findings is the presence of abundant desmoplastic stroma in the tumor, which resembles scirrhous carcinoma in various organs, such as the stomach and colon [23]. In cases with IMCC, DWI usually showed a target sign. Kajiyama et al*.* [24] reported that patient with scirrhous-type ICC had a poorer prognosis due to more frequent lymphatic permeation and perineural invasion and higher proliferative activity of tumor cells when compared with non-scirrhous ICC. In our study, the high-expression group was more commonly shown with a target sign (69.2%), suggesting a poor prognosis in this group, which is consistent with the previous studies. Furthermore, the biological importance and clinical effect of the desmoplastic stroma in cholangiocarcinoma have only been recently understood. There is now increasing evidence to suggest that the desmoplastic reaction at the cellular and molecular levels has a crucial role in promoting enhanced malignant behavior and therapeutic resistance in patients with cholangiocarcinoma.

The HBP enhancement pattern of the low-expression group also differed from the high-expression group. Low-expression group manifested mostly as cloud signs, while the high-expression group was dominated by partially hyperintense [6]. “Cloud sign” is a special, but not specific, pattern in the HPB image suggesting IMCC. HBP is expected to be seen in adenocarcinoma patients with metastases. It has been reported that the pathological basis of IMCC for cloud sign on HBP is the large interstitial spaces seen in stromal fibrosis that may retain contrast agents [25]. Based on these data, we expected to see more cloud signs in HBP in the high-expression group; still, the opposite results were obtained (more signs were seen in the low-expression group). This may be explained as follows: firstly, there was a statistically significant difference in the diameter of the two groups; the average diameter in the low-expression group was 3.69 cm vs. 7.96 cm in the high-expression group. The contrast agent that infiltrates into the center interstitial spaces of the stromal fibrosis needs more time to infiltrate a large tumor. In this study, we obtained HBP images 10–15 min after injection, which might affect the results (time too short). Mamone et al*.* [26] suggested that the partially hyperintense group in the clinical HBP phase may have more fibrosis just because of the insufficient time for the MRI. Thus, more prospective studies are needed to confirm this hypothesis. Secondly, the low hypointense portion on HBP of the high expression group may be attributed to necrosis rather than lack of fibrosis [26, 27]. Kang et al*.* [28] found that medium-differentiated IMCC has a significantly higher percentage of relative enhancement on hepatobiliary phase images compared to low differentiated IMCC, which contains more tumor necrosis. The high-expression Ki-67 group with lower differentiated IMCC may contribute to more tumor necrosis and cause hypointensity in HBP.

This study has a few limitations. First, this was a retrospective study with small sample size. Second, although we used standardized histological examination procedures and two experienced pathologists provided detailed pathology reports, matching MR images of specific tumors and histopathological results were challenging. Fortunately, previous studies have provided evidence to prove a histopathological interpretation of IMCC signs. Thus, further study with a larger, prospectively collected population with long follow-up is warranted to confirm these results.To obtain more information on histopathology prospectively, such as microvascular and lymphatic invasion, which is closely related to the postoperative prognosis of patients, combined with gadoxetic acid-enhanced MRI will surely provide more comprehensive and valuable information for the diagnosis and treatment of IMCC.

In conclusion, the nomogram consisting of DWI and HBP could be a useful predictive method for the Ki-67 expression of IMCC.

## Supplementary Information

Below is the link to the electronic supplementary material.

Supplementary file1 (DOCX 721 kb)

## Abbreviations

IMCC: Intrahepatic mass cholangiocarcinoma
DCA: Decision curve analysis
ICC: Intrahepatic cholangiocarcinoma
HCC: Hepatocellular carcinoma
MF: Mass-forming
PI: Periductal infiltrating
IG: Intraductal growing
IMCC: Intrahepatic mass-forming cholangiocarcinoma
OS: Overall survival
AFP: Alpha-fetoprotein
CEA: Carcinoembryonic antigen
CA19-9: Carbohydrate antigen 19–9
Gd‐EOB‐DTPA: Gadolinium-ethoxybenzyl-diethylenetriamine pentaacetic acid
DWI: Diffusion-weighted imaging
T1WI: T1-weighted imaging
T2WI: T2-weighted imaging
HBP: Hepatobiliary phase
SIR-HBP: The signal ratio of lesion-liver parenchyma on hepatobiliary phase
ROI: Regions of interest
SI: The signal intensity
ICCs: The intraclass correlation coefficients
AUC: The area under curve
