# Supplementary material for: Nomogram based on MRI for preoperative prediction of Ki-67 expression in patients with intrahepatic mass cholangiocarcinoma
Source: Abdom Radiol (NY). 2022 Nov 19;48(2):567–78. doi: 10.1007/s00261-022-03719-7 (PMC9902416; doi:10.1007/s00261-022-03719-7)
Supplement: Supplementary file 1 — Supplementary file1 (DOCX 721 kb) [file 261_2022_3719_MOESM1_ESM.docx]

**Nomogram based on MRI for Preoperative Prediction of Ki-67 Expression in Patients with Intrahepatic Mass Cholangiocarcinoma**

**journal name:** **Abdominal Radiology**

**Author Names/Affiliations and Corresponding Author:**Xiang Chen^1†^、Jingfen Zhu^1†^、Zigui Zou^2†^、Mingzhan Du^2^、Junjian Xie^1^、Yujie Ye^1^、Ling Zhang^3,4,5^*、Yonggang Li^1,6,7^*

†These authors contributed equally to this work and share first authorship.

1. Department of Radiology, the First Affiliated Hospital of Soochow University, Suzhou city, Jiangsu province, P.R. China 215000

2. Department of Pathology, the First Affiliated Hospital of Soochow University, Suzhou city, Jiangsu province, P.R. China 215000

3. Department of Radiology, Sun Yat-sen University Cancer Center, Guangzhou city, Guangdong province, P.R. China 510060

4. State Key Laboratory of Oncology in South China, Guangzhou city, Guangdong province, P.R. China 510060

5. Collaborative Innovation Center for Cancer Medicine, Guangzhou city, Guangdong province, P.R. China 510060

6. National Clinical Research Center for Hematologic Diseases, the First Affiliated Hospital of Soochow University, Suzhou city, Jiangsu province, P.R. China 215000

7. Institute of Medical Imaging, Soochow University, Suzhou city, Jiangsu province, P.R. China 215000

^*^**Corresponding authors**:

1. Yonggang Li, Shizi Street 188#, Suzhou city, Jiangsu province, P.R. China 215000, e-mail: [liyonggang224@163.com](mailto:liyonggang224@163.com), telephone number: +86 13776006850

2. Ling Zhang, Dongfeng East Road 651#, Guangzhou city, Guangdong province, P.R. China 510060, e-mail: [zhangl@sysucc.org.cn](mailto:zhangl@sysucc.org.cn)

**Supplementary Appendix 1: MRI protocols and the detailed parameters of the MR sequences**

MR images were acquired using a 3.0-T MRI scanner (Magnetom Verio; Siemens Healthcare, Erlangen, Germany) with a 16-channel phase-array coil that covered the whole liver. The MR scan sequences were as follows: 1) a three-dimensional volume interpolated breath-hold examination (3D VIBE) T1-weighted in-phase with time of repetition (TR) = 4.16 ms, time of echo (TE) = 2.58 ms, field of view (FOV) = 26 cm × 32 cm and slice thickness = 5 mm; 2) a 3D VIBE T1-weighted out-phase with TR = 4.16 ms, TE = 1.35 ms , FOV = 26 cm × 32 cm and slice thickness = 5 mm; 3) a respiration-triggered T2-weighted fat-suppression turbo spin-echo with TR = 3, 920-7, 345 ms, TE = 105 ms, FOV = 26 cm × 32 cm and slice thickness = 6 mm; 4) diffusion-weighted imaging (DWI, b = 0 or 50, 800 sec/mm^2^) with a free-breathing single-shot echo-planar technique and TR = 5,300 ms, TE = 57 ms, field of view (FOV) = 26 cm × 32 cm and slice thickness = 8 mm.

A food-fast of more than 6 hours and water-fast of more than 4 hours before scanning were required for all patients. The dynamic enhancement was also performed with the 3D VIBE T1-weighted imaging fat saturation sequence with TR = 3.90 ms, TE = 1.89 ms, FOV = 26 cm × 32 cm, and slice thickness =3 mm. A dose of 25 μmol per kg of body weight of Gd-EOB-DTPA (Primovist, Bayer Schering Pharma, AG, Berlin, Germany) was injected as a rapid bolus and was immediately followed by 30 ml saline at a rate of 1 ml/s. The images in arterial phases (AP), portal venous phases (PVP) and transitional phase (TP) were obtained during suspended respiration at 30-35 sec, 65-70 sec, and 180-185 sec, respectively. After injection of Gd-EOB-DTPA, HBP images were obtained at 20 min. AP, PVP, HBP and T2W images were retrieved from a picture archiving and communication system (PACS, Neusoft Version 5.5).

For patients (n=53) who received Gd-EOB-DTPA (Eovist/Primovist, Bayer Healthcare), delayed hepatobiliary phase (HBP) imaging was obtained at 15 and 20 min post-contrast injection. The remaining 11 patients received extra-cellular contrast material (ECCM): gadopentetate dimeglumine (Magnevist, Bayer Healthcare), gadobutrol (Gadavist, Bayer Healthcare) or gadobenate dimeglumine (MultiHance, Bracco Imaging).

**Supplementary Appendix 2**

| The inter reader agreement for any of the qualitative MRI features | | | | |  |
| --- | --- | --- | --- | --- | --- |
|  | value | Asymptotic  Standardized  Error^a^ | Approximate  T^b^ | Approximate  significance |  |
| Location | 0.884 | 0.049 | 10.272 | 0.000 |  |
| Margin | 0.785 | 0.103 | 6.613 | 0.000 |  |
| Contour | 0.927 | 0.042 | 9.807 | 0.000 |  |
| Satellite lesions | 0.942 | 0.041 | 7.836 | 0.000 |  |
| Intrahepatic metastasis | 0.855 | 0.063 | 7.102 | 0.000 |  |
| Biliary dilation | 0.825 | 0.068 | 6.854 | 0.000 |  |
| Capsular retraction | 0.796 | 0.073 | 6.612 | 0.000 |  |
| Ascites | 0.880 | 0.058 | 7.322 | 0.000 |  |
| Arterial phase enhancement pattern | 0.908 | 0.045 | 10.465 | 0.000 |  |
| Dynamic enhancement pattern | 0.885 | 0.050 | 9.930 | 0.000 |  |
| HBP enhancement pattern | 0.893 | 0.052 | 8.470 | 0.000 |  |
| Abnormal perfusion | 0.948 | 0.052 | 7.884 | 0.000 |  |
| Halo sign | 0.905 | 0.066 | 7.519 | 0.000 |  |
| peritumoral hypointense on HBP | 0.853 | 0.063 | 7.090 | 0.000 |  |
| T1WI | 0.942 | 0.041 | 7.836 | 0.000 |  |
| T2WI | 0.948 | 0.052 | 7.884 | 0.000 |  |
| DWI | 0.855 | 0.062 | 7.105 | 0.000 |  |
| *IMCC，intrahepatic mass cholangiocarcinoma ;MRI, Magnetic Resonance Imaging ; T1WI，T1-weighted imaging; T2WI, T2-weighted imaging; CE, contrast enhancement; HBP, hepatobiliary phase; DWI, diffusion-weighted imaging | | | | | |

**Supplementary Appendix 3**

| The interclass correlation coefficient for Quantitative MRI features | | | | |  |
| --- | --- | --- | --- | --- | --- |
|  |  | Intraclass correlation |  | 95% confidence interval | |
| SIR-HBP | Single measures | 0.995 |  | 0.991 | 0.997 |
|  | Average measures | 0.997 |  | 0.995 | 0.998 |
| Diameter | Single measures | 0.969 |  | 0.947 | 0.982 |
|  | Average measures | 0.984 |  | 0.973 | 0.991 |
| ADC_mean_ | Single measures | 0.978 |  | 0.934 | 0.990 |
|  | Average measures | 0.989 |  | 0.966 | 0.995 |
| nADC_mean_ | Single measures | 0.972 |  | 0.953 | 0.984 |
|  | Average measures | 0.986 |  | 0.976 | 0.992 |

**Supplementary Appendix 4**

| Results of multivariate logistics analysis for predicting Ki-67 expression in IMCC | | | | |
| --- | --- | --- | --- | --- |
| Model | Predictors | *P* value | OR | 95%CI |
| Clinical model | chronic hepatitis | 0.020 | 0.253 | 0.080-0.806 |
| MRI model | Diameter | 0.029 | 3.236 | 1.127-9.290 |
|  | DWI | 0.017 | 1821.227 | 3.747-885176.094 |
| Combined model | DWI | 0.010 | 1822.741 | 6.189-536781.805 |
|  | HBP enhancement pattern | 0.046 | 14.270 | 1.044-195.039 |
| MRI, Magnetic Resonance Imaging; DWI, diffusion-weighted imaging; HBP, hepatobiliary phase; OR, odds ratio; CI, confidence interval; IMCC, intrahepatic mass cholangiocarcinoma | | | | |


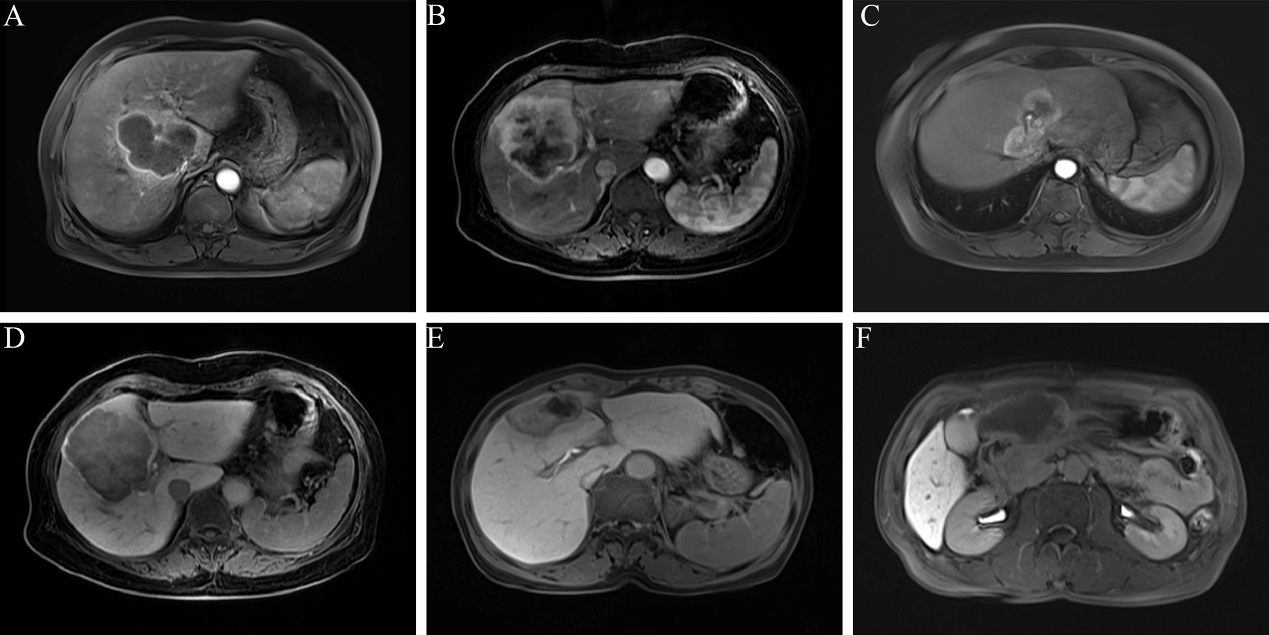


**Supplementary figure 1** Arterial phase enhancement patterns**(A-C)**. **(A)** peripherally hyperintense: <10% of the largest tumor diameter is hyperintense; **(B)** partially hyperintense: 10%–70% of the largest tumor diameter is hyperintense; **(C)** Diffusely hyperintense: more than 70% of the largest tumor diameter is hyperintense.The hepatobiliary phase (HBP) enhancement pattern**(D-F)**. **(D)**cloud sign: a central hyperintense area with a peripheral hypointense rim defect, ＞90% of the largest tumor diameter is hyperintense; **(E)** partially hyperintense signal: 10%–90% of the largest tumor diameter is hyperintense; **(F)** peripherally hyperintense signal: <10% of the largest tumor diameter is hyperintense.
